# Combined Analysis of Murine and Human Microarrays and ChIP Analysis Reveals Genes Associated with the Ability of MYC To Maintain Tumorigenesis

**DOI:** 10.1371/journal.pgen.1000090

**Published:** 2008-06-06

**Authors:** Chi-Hwa Wu, Debashis Sahoo, Constadina Arvanitis, Nicole Bradon, David L. Dill, Dean W. Felsher

**Affiliations:** 1 Departments of Medicine and Pathology, Division of Oncology, Stanford University School of Medicine, Stanford, California, United States of America; 2 Department of Electrical Engineering, Stanford University, Stanford, California, United States of America; 3 Department of Computer Science, Stanford University, Stanford, California, United States of America; University of Pennsylvania, United States of America

## Abstract

The MYC oncogene has been implicated in the regulation of up to thousands of genes involved in many cellular programs including proliferation, growth, differentiation, self-renewal, and apoptosis. MYC is thought to induce cancer through an exaggerated effect on these physiologic programs. Which of these genes are responsible for the ability of MYC to initiate and/or maintain tumorigenesis is not clear. Previously, we have shown that upon brief MYC inactivation, some tumors undergo sustained regression. Here we demonstrate that upon MYC inactivation there are global permanent changes in gene expression detected by microarray analysis. By applying StepMiner analysis, we identified genes whose expression most strongly correlated with the ability of MYC to induce a neoplastic state. Notably, genes were identified that exhibited permanent changes in mRNA expression upon MYC inactivation. Importantly, permanent changes in gene expression could be shown by chromatin immunoprecipitation (ChIP) to be associated with permanent changes in the ability of MYC to bind to the promoter regions. Our list of candidate genes associated with tumor maintenance was further refined by comparing our analysis with other published results to generate a gene signature associated with MYC-induced tumorigenesis in mice. To validate the role of gene signatures associated with MYC in human tumorigenesis, we examined the expression of human homologs in 273 published human lymphoma microarray datasets in Affymetrix U133A format. One large functional group of these genes included the ribosomal structural proteins. In addition, we identified a group of genes involved in a diverse array of cellular functions including: BZW2, H2AFY, SFRS3, NAP1L1, NOLA2, UBE2D2, CCNG1, LIFR, FABP3, and EDG1. Hence, through our analysis of gene expression in murine tumor models and human lymphomas, we have identified a novel gene signature correlated with the ability of MYC to maintain tumorigenesis.

## Author Summary

The targeted inactivation of oncogenes may be a specific and effective treatment of cancer. However, how oncogene inactivation leads to tumor regression is not clear. Previously, we have shown that even the brief inactivation of the MYC oncogene can result in the sustained regression of at least some tumors. To understand the mechanism, we have utilized several novel genomic analyses to define a set of genes that strongly correlate with the ability of the MYC oncogene to maintain tumorigenesis. First, we generated a novel data set from microarray analyses of murine tumors that we analyzed by StepMiner to identify discrete step changes in gene expression after the inactivation or the reactivation of the MYC oncogene. Second, we utilized Boolean Network Analysis to further define the subset of genes highly correlated with MYC in human tumorigenesis. Third, we utilized ChIP analysis to demonstrate that in many cases the permanent changes of gene expression we uncovered were associated with changes in the ability of MYC to occupy the promoter locus. Our general strategy could be similarly utilized in other experimental model systems to understand how specific oncogenes contribute to the maintenance of tumorigenesis.

## Introduction

Overexpression of MYC is one of the most frequent events in human tumorigenesis [1]. MYC overexpression is thought to induce tumorigenesis by causing inappropriate gene expression resulting in autonomous cellular growth, proliferation, and the inhibition of cellular differentiation [2],[3]. Many laboratories have conditionally overexpressed c-MYC (MYC) utilizing conditional transgenic model systems [4]–[9]. In these models, the suppression of MYC led to permanent loss of tumorigenesis through proliferative arrest, differentiation and/or apoptosis [4]–[6],[10]. In some circumstances, even the brief suppression of MYC overexpression permanently prevents its ability to sustain tumorigenesis [6]. These and other observations have suggested the possibility that oncogenes such as MYC exhibit the phenomena of oncogene addiction [11]. However, the molecular basis of oncogene addiction is not clear. Recently, we have suggested that cellular senescence, which involves chromatin modifications and heterochromatin formation [12],[13], may be an important mechanism for sustained tumor regression upon MYC inactivation [14].

MYC is thought to play a role in the regulation of up to 15% of genes in the fly, mouse or human [3],[15],[16]. Thus, it seems likely that changes in gene expression programs, rather than individual genes, account for the phenotypic consequences of MYC inactivation. Consistent with this notion, MYC has recently been shown to globally influence chromatin structure through histone modifications [17]–[19]. Similarly, N-MYC was shown to globally regulate acetylation and methylation of histone molecules [20]. We have reported that MYC inactivation in tumors induces specific global changes in histone modification [14]. Although many MYC target genes have been identified in various cells or tissue contexts (summarized in http://www.myc-cancer-gene.org), it is hard to discern which of the many of MYC targets are associated with the ability of MYC to initiate and/or sustain tumorigenesis.

**Figure 1:**
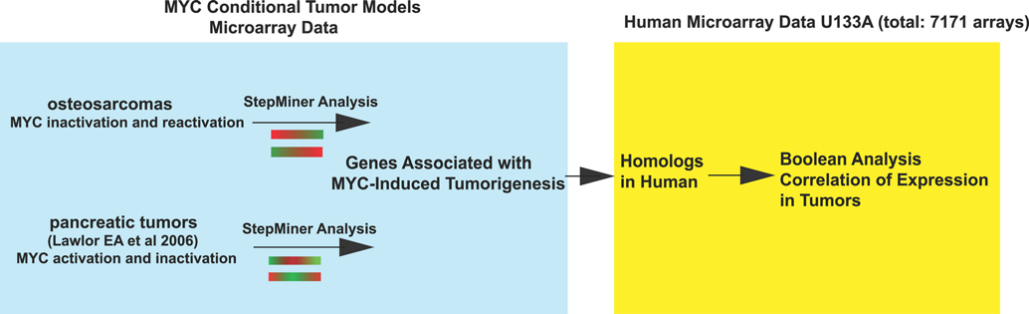
Overview of our general strategy to define a gene signature associated with the ability of MYC to initiate and maintain tumorigenesis.

Many previous studies have examined changes in gene expression associated with the induction of MYC expression in cells [3], [15], [21]–[26]. Other groups have performed comparative analysis of gene expression profiles between murine constitutive MYC-induced tumors and human tumors in liver and prostate cancers [27],[28]. Both of these analyses identified similarities in gene expression between MYC-induced tumor models and human tumors. Although revealing, these studies would not necessarily identify gene products that are responsible for the ability of MYC to induce tumorigenesis. We speculated that by analyzing gene expression profiles in tumors generated from conditional transgenic models would allow us to identify gene expression signature specifically associated with the ability of MYC to initiate and maintain tumorigenesis. We performed microarrays on mRNA samples from a time-course experiment with MYC inactivated and then reactivated in osteosarcoma. The expression data was then examined using the StepMiner algorithm [29] to generate a list of genes associated with MYC-induced tumorigenesis in osteosarcomas (Figure 1). The StepMiner algorithm analyzes microarray time courses by identifying genes that undergo abrupt transitions in expression level, and the time at which the transitions occur. Importantly, by ChIP we were able to demonstrate that permanent changes in gene expression were frequently associated with measurable alterations in the ability of MYC to bind to the promoter regions of these genes in osteosarcomas. Furthermore, gene expression profiles were compared between osteosarcomas and the previously published MYC conditional pancreatic tumor [25] to generate a common gene signature associated with MYC-induced tumorigenesis in mice (Figure 1). Finally, Boolean analysis was used to further examine the correlation between levels of expression of this identified subset of genes among the published dataset of 7,171 human microarrays in U133A format. From this analysis, we were able to deduce a list of genes strongly correlated with the ability of MYC to maintain tumorigenesis.

## Results

### MYC Inactivation and Reactivation Induces Global Changes in Gene Expression Identified by the StepMiner Algorithm

**Figure 2:**
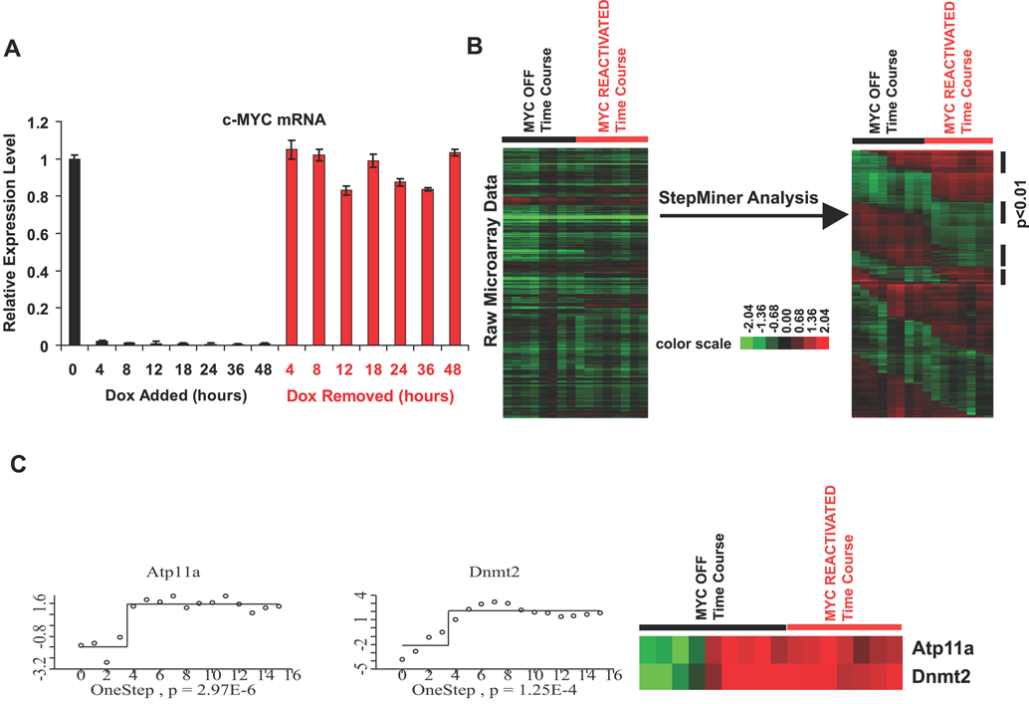
Microarray data analysis for gene expression in osteosarcomas upon MYC inactivation and reactivation by the StepMiner algorithm. (A) Quantitative Real-time PCRs for MYC Transgene Expression (Human cMYC) upon treatments and removals of doxycycline in Bone Tumor Cells. Bone tumor cells 1325 were treated with 20ng/ml of doxycycline for 0, 4, 8, 12, 18, 24, 36 and 48 hours before collecting total RNA. Excess amount of PBS was applied to cells treated with doxycyline for 48 hours to remove doxycycline. At 4, 8, 12, 18, 24, 36 and 48 hours after removing doxycycline, cells were collected for total RNA by Trizol and cDNA was synthesized by Superscript II. cDNA templates from all these samples were used for quantitative real-time PCR with fluorescence labeled human c-MYC and mouse GAPDH probes to validate MYC expression. MYC expression at each time point was normalized with the MYC expression in the original tumor before doxycycline treatment. (B) Raw data from the microarray experiments at different time points was applied with StepMiner analysis to find the patterns of interest (expression went down upon MYC inactivation and stayed down upon MYC reactivation, expression went up upon MYC inactivation and stayed up upon MYC reactivation). p<0.01 was set to be the cutoff for significant changes among experiments. (C) Examples of StepMiner analysis are shown. In left panels, x-axis is the array number (MYC inactivation for 0 hour = 1, 4 hour = 2, 8 hour = 3, 12 hour = 4, 18 hour = 5, 24 hour = 6, 36 hour = 7, 48 hour = 8, MYC reactivation for 4 hour = 9, 8 hour = 10, 12 hour = 11, 18 hour = 12, 24 hour = 13, 36 hour = 14, 48 hour = 15. ) and y-axis is the level of mRNA expression compared with reference RNA. Typical one step changes are listed for Atp11a and Dnmt2 (p = 2.97×10^−6^ and p = 1.25×10^−4^ expression went up upon MYC inactivation and stayed up for MYC reactivation). Centering the data with the expression level at the identified step was applied for illustration purposes.

The MYC induced osteosarcoma derived cell line, 1325, was grown *in vitro* [6] and treated with 20ng/ml of doxycycline in complete DMEM medium for various length of time to inactivate MYC expression. To reactivate MYC expression, doxycyline was removed by rinsing the bone tumor cells with an excess amount of PBS. mRNA was collected from bone tumor cells treated with doxycycline for 0, 4, 8, 12, 18, 24, 36, and 48 hours, and after removal of doxycycline for 4 , 8, 12, 18, 24, 36, and 48 hours. MYC levels were greatly reduced as early as 4 hours after doxycycline treatment (Figure 2A and Figure S1). We confirmed that the expression of MYC could be reactivated to a level similar to that of MYC-on tumors by thoroughly washing the cells with PBS (Figure 2A and Figure S1).

cDNA microarray analysis was performed on the RNA samples prepared from tumors in which MYC was inactivated and reactivated for different lengths of time. StepMiner analysis (Figure 2B, 2C) [29] was applied to this time-course microarray experiment to identify changes in gene expression at discrete time points before and after MYC inactivation and reactivation. StepMiner fits step functions to the data points using an adaptive regression scheme and identifies time points at which a gene is significantly induced or repressed. Examples of one-step expression pattern are illustrated in Figure 2C.

Recently, we have shown that MYC inactivation generally induces cellular senescence in several tumor models [14]. Therefore, we specifically examined if the expression of senescence associated genes changed upon MYC inactivation in osteosarcomas. Indeed, we did find that senescence associated genes such as p15INK4b, p21CIP, PCNA, MCM3, CYCLIN A [12],[30],[31] were up-regulated or down-regulated upon MYC inactivation (Figure S2 and Table S1). Thus, our results support the notion that MYC inactivation is inducing changes in gene expression that is associated with cellular senescence.

**Figure 3:**
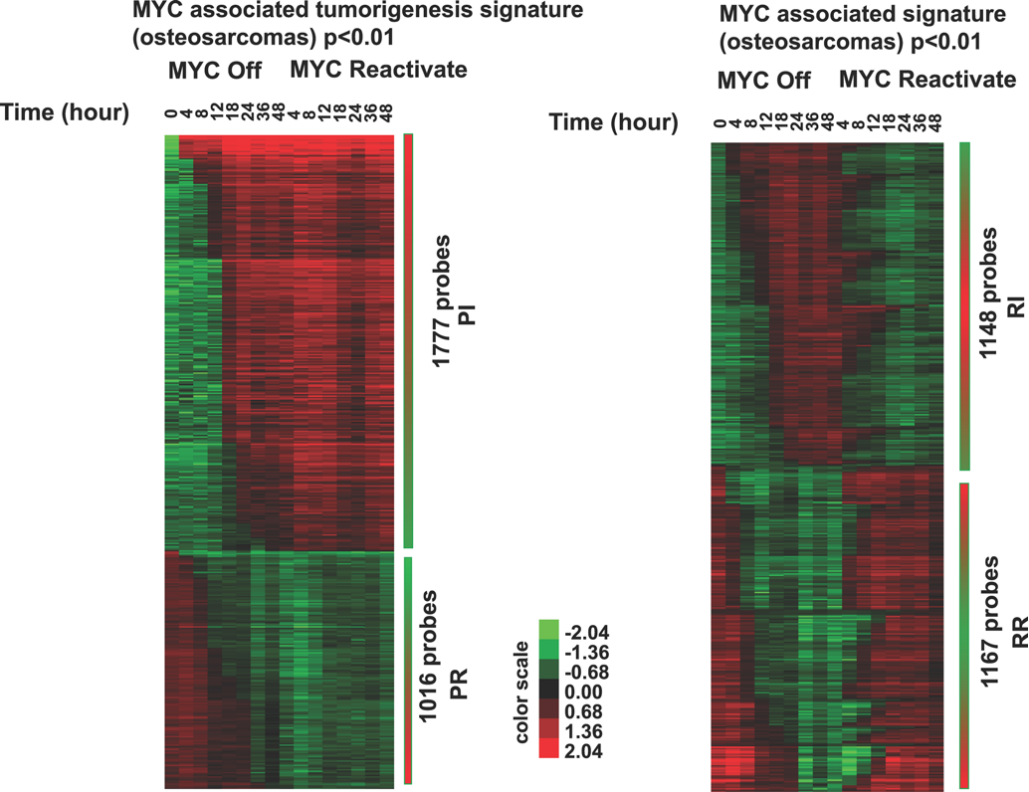
Permanent and reversible changes of gene expression upon MYC inactivation in osteosarcomas identified by the StepMiner algorithm. Statistically significant changes (p<0.01) with specific patterns are listed here: 1777 probes with expression went up after MYC inactivation and stayed up upon MYC reactivation (the PI group), 1016 probes with expression went down after MYC inactivation and remained down upon MYC reactivation (the PR group), 1148 probes with expression went up after MYC inactivation and went back down upon MYC reactivation (the RI group) and 1167 probes with expression went down after MYC inactivation and went back up upon MYC reactivation (the RR group). Lists of gene names in each group are shown in Table S2. Pie Charts summarizing numbers of genes in each group are shown in supplementary Figure 3.

Generally, analysis of gene expression changes after StepMiner analysis revealed four discrete patterns of changes in gene expression upon MYC inactivation and reactivation: Permanently Repressed (PR), Permanently Induced (PI), Reversibly Repressed (RR) and Reversibly Induced (RI). For this analysis, we set p<0.01 as a cutoff for statistically significant changes in gene expression. We identified 1016 unique probes in the PR group, 1777 unique probes in the PI group, 1148 unique probes in the RI group, and 1167 unique probes in the RR group (Figure 3 and Tables S2 for lists of genes).

Based upon our previously published observation that even brief inactivation of MYC can result in the sustained loss of the neoplastic properties of MYC-induced osteosarcomas [6], we speculated that genes which are potentially important for sustained tumorigenesis would be permanently repressed or induced (e.g. the PR group or the PI group) upon MYC inactivation.

To identify associated functional activities associated with the PR and PI groups of genes, we applied Gene Ontology analysis (GO Term analysis) to the list of genes generated above. Biological functions that were identified for each step upon MYC inactivation are listed (Table S3, S4, and S5). Associated functions identified include gene products known to regulate metabolism, biosynthesis of nucleotides and proteins and genes involved in the regulation or function of ribonucleoprotein complexes.

**Figure 4:**
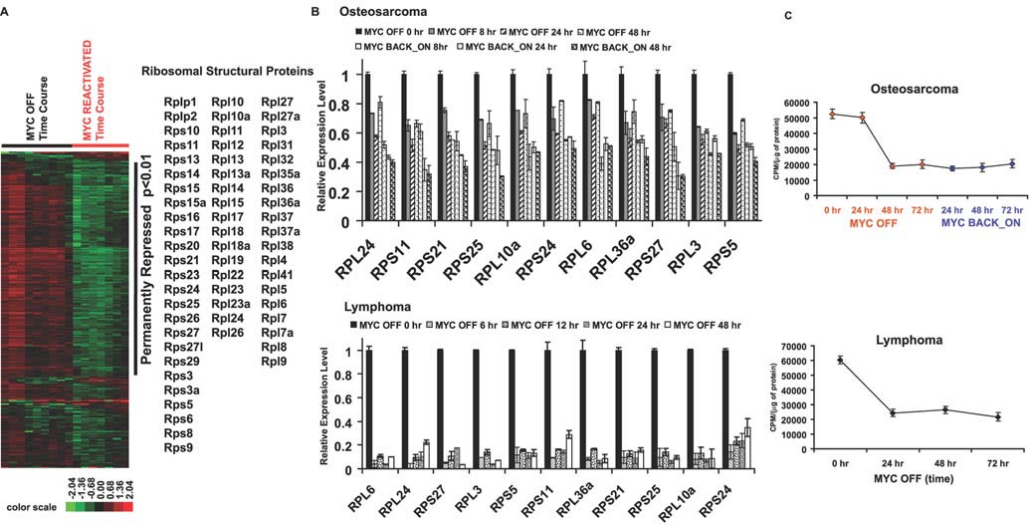
mRNA expression of ribosomal structural proteins and the associated rates of protein synthesis were reduced upon MYC inactivation in osteosarcomas and lymphomas. (A) Gene list of all the ribosomal structural proteins (total of 82 genes) were generated and applied for filtering the microarray data of MYC inactivation and reactivation time-course. 61 ribosomal structural proteins with p<0.01 in StepMiner analysis are shown on the right. (B) validation of mRNA expression of ribosomal structural proteins RPS5, RPS11, RPS21, RPS24, RPS25, RPS27, RPL3, RPL6, RPL10a, RPL24 and RPL44 by quantitative real-time PCR in osteosarcomas upon MYC inactivation and reactivation (top panel) and lymphomas upon MYC inactivation (bottom panel). (C) Rates of protein synthesis upon MYC inactivation (bone tumors and lymphomas) and reactivation (bone tumors) were assayed by measuring the S^35^ methione and S^35^ Cysteine incorporation to the protein.

Notably, MYC has been shown to regulate expression of ribosomal structure proteins and ribosomal RNAs [18],[32]. Hence, it is striking that the mRNA expression of 61 ribosomal structural proteins out of 82 ribosomal structural protein genes was decreased upon MYC inactivation and further decreased upon MYC reactivation in bone tumor (see Figure 4A and Table S3 for results of GO term analysis). To validate that these genes expression did change, we performed quantitative real-time PCR of 11 ribosomal structural proteins in osteosarcomas (Figure 4B). Moreover, we found that the same ribosomal structural proteins also changed upon MYC inactivation in our conditional model of lymphomas [4] (Figure 4B). We then examined if the decreased expression of ribosomal structural proteins associated with changes in rate of protein synthesis. We found that the protein synthesis rates were decreased in both bone tumor and lymphomas upon MYC inactivation (Figure 4C). Furthermore, the protein synthesis rate remained lowed upon MYC reactivation in bone tumor (Figure 4C).

**Figure 5:**
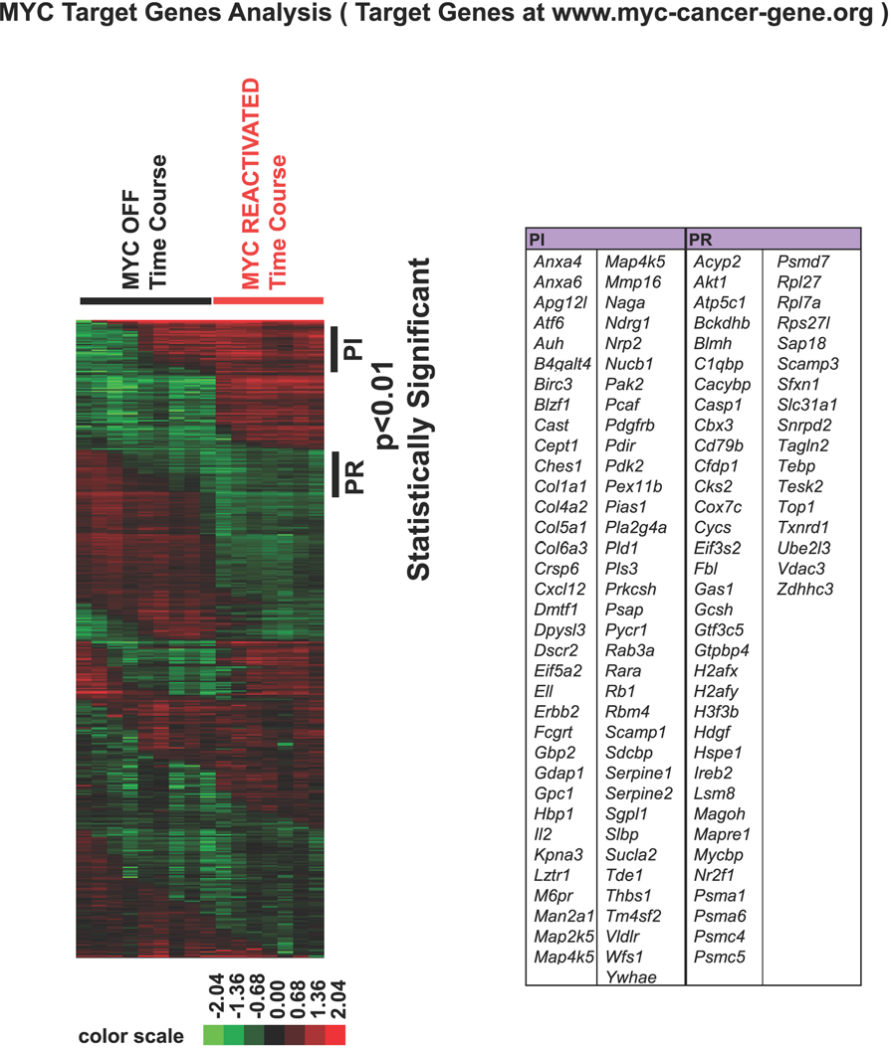
Expression of previously published MYC targets was permanently repressed or induced upon MYC inactivation in osteosarcomas. MYC targets collected in www.myc-cancer-gene.org were used as a list to filter the microarray data from osteosarcomas upon MYC inactivation and reactivation. Gene expression permanently repressed (PR) or permanently induced (PI) were identified while MYC was inactivated in bone tumors.

**Figure 6:**
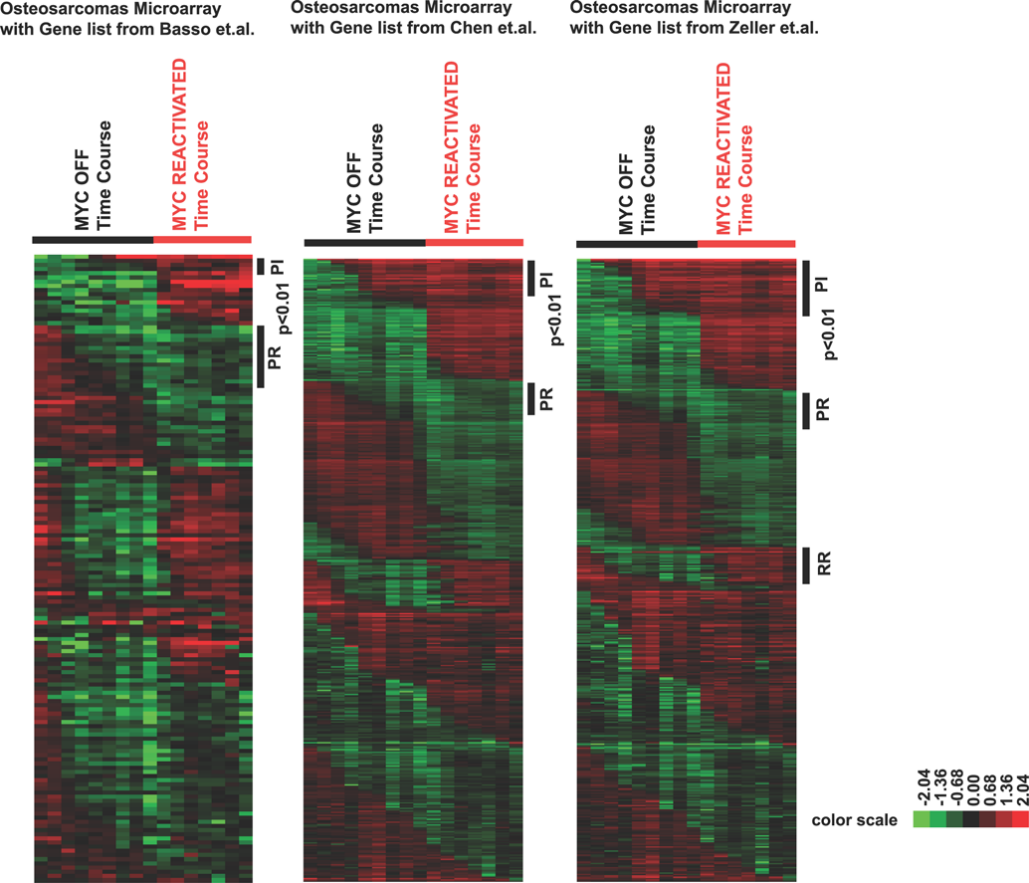
Expression of previously published direct MYC targets was permanently repressed or induced upon MYC inactivation in osteosarcomas. Murine homologs of directed MYC targets identified from 3 publications [33]–[35] were retrieved and their expression in the bone tumors upon MYC inactivation and reactivation was examined. Genes scored statistically significant (p<0.01) in the StepMiner analysis were labeled.

MYC has been shown to regulate the gene expression of a multitude of genes [3], [15], [21]–[26], [33]–[35]. To examine if these genes changed in gene expression upon MYC inactivation and reactivation, we used two approaches. First, we retrieved the mouse homologs of MYC target genes listed in www.myc-cancer-gene.org, a collection of most of the published MYC target genes in different organisms and tissues (total of 1697 MYC targets)[16]. In osteosarcomas, 71 of the published MYC targets are permanently induced and 52 of the published MYC targets are permanently repressed upon MYC inactivation and reactivation (p<0.01) (Figure 5, see PR and PI). Second, we examined direct MYC target genes identified as defined by several recent publications [33]–[35]. Interestingly, only 7–11% of these identified direct MYC target genes exhibited sustained changes upon MYC inactivation in osteosarcoma (Figure 6 and Table S6).

### MYC Binding to Promoter Loci Correlated with Permanent Changes in Gene Expression

An important recent report suggests that MYC binding to promoters is regulated by the chromatin structure at these gene loci [36]. Recently, we have shown that MYC inactivation is associated with global changes in chromatin structures [14]. Thus, it seemed that a possible explanation for the permanent changes in gene expression that we observed (Figure 3) is that the ability of MYC to bind to specific gene products is perturbed by changes in chromatin structure. To address this possibility directly, we used ChIP to examine MYC binding to E-box sequences of target genes in MYC activated and MYC reactivated conditions for osteosarcomas.

**Figure 7:**
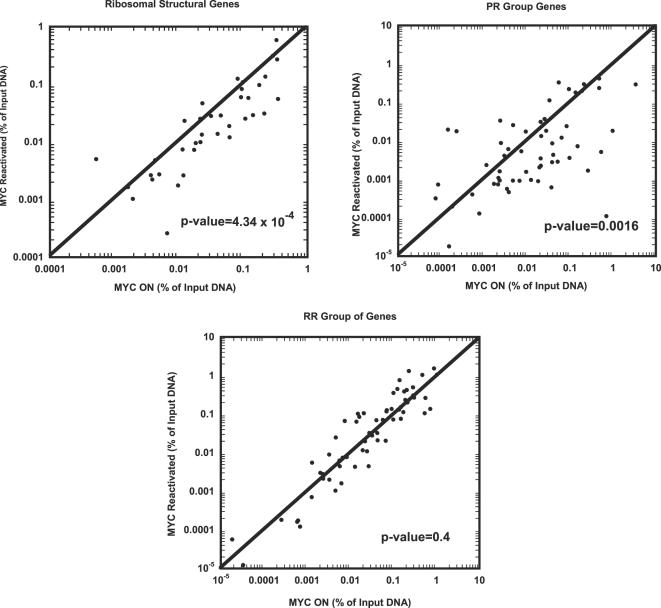
Changes of MYC binding to E-box regions for genes permanently repressed upon MYC inactivation. ChIP for MYC binding to E-box regions were examined for ribosomal structural proteins (Figure 4) and genes with the PR pattern and RR pattern expression whose homologs were shown to be direct MYC targets before (Figure 6 and [35]). Dot plots with percentages of DNA relative to input DNA brought down by the MYC antibody for MYC ON and MYC reactivated (48 hours) conditions in bone tumor cells are shown here (see Table S7 for the raw data, average of two experiments). The p-values shown here were calculated by Z-test.

We specifically examine three groups of genes: the ribosomal structural proteins (Figure 4), the PR group (Figure 6, [35]) and the RR group genes that were identified previously as direct MYC targets before (Figure 6, [35]). A total of 168 E-box regions were examined by ChIP. As a control, we performed ChIP for osteosarcoma in the MYC OFF condition (Table S7). Binding of MYC to E-box regions is shown as the percentage of DNA brought down by ChIP for the MYC ON *versus* the MYC reactivated conditions (Figure 7). Note, that upon MYC reactivation the majority of ribosomal structural genes exhibited decreased MYC binding to E-boxes relative to the MYC ON condition (31 out of 41 data points fall below the line of X = Y, p-value = 4.34×10^−4^). Similarly, the majority of the genes with the PR pattern of gene expression exhibited a significant decrease of MYC binding to E-boxes relative to the MYC ON condition (42 out of 60 data points fall below the line of X = Y, p-value = 0.0016) when MYC was reactivated (Figure 7 and Table S7). In contrast, the group of genes that exhibited the RR pattern of gene expression exhibited no particular increase or decrease in MYC binding to E-boxes compared with the MYC ON condition (33 out of 67 data points fall below the line of X = Y, p-value = 0.4). Our results support the possibility that the permanent changes in gene expression upon MYC inactivation can be explained in many cases because of a change in the ability of MYC to bind to specific promoter loci.

### Comparative Analysis of Osteosarcoma Microarray Data to a Previously Published Microarray Dataset

To determine if the gene signature we identified would also be seen in another tumor model system, we compared our microarray data from MYC-induced osteosarcoma with a previously reported microarray data set from a MYC-induced pancreatic tumor model to identify a common expression signature for MYC-induced tumorigenesis [25]. In the published report, MYC-ER^TAM^ was expressed specifically in β-cell pancreatic tissues with MYC-on for 2, 4, 8, 24 hours, and 21 days (referred as tumorigenesis arrays in the published paper), and MYC off in pancreatic tumors for 2, 4, and 6 days (referred as tumor regression arrays in the published paper). MYC activation induced pancreatic tumors and MYC inactivation resulted in tumor regression through apoptosis [7]. cDNA from these samples was applied to oligo arrays from Affymetrix [25]. As previously suggested in the paper, we assumed that genes were induced (repressed) upon MYC activation and repressed (induced) upon MYC inactivation were potentially important for MYC induced tumorigenesis.

**Figure 8:**
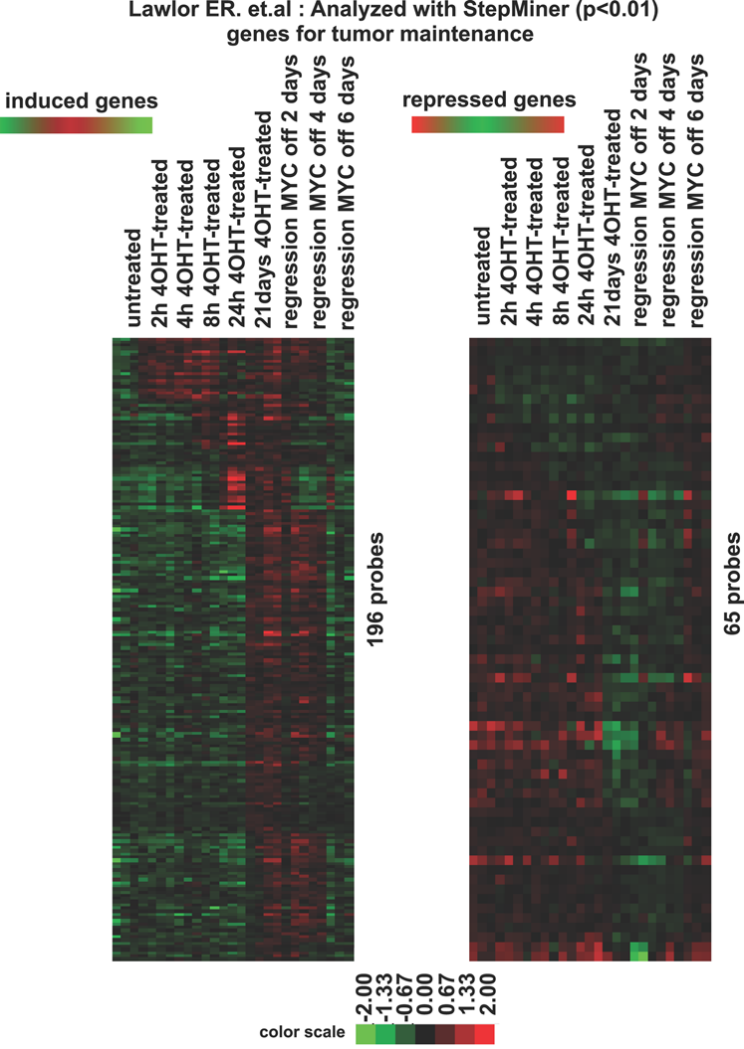
Genes associated with MYC-induced tumorigenesis in a MYC-induced pancreatic tumor model identified by the StepMiner algorithm. Microarray data of the time-course experiments from a MYC-induced pancreatic tumor model [25] was retrieved and the StepMiner algorithm was applied to the data to identify genes whose expression went up in tumor initiation and came down in tumor regression (induced genes) and genes whose expression went down in tumor initiation and came back up in tumor regression (repressed genes) as potential genes associated with MYC induced tumorigenesis in pancreatic tumors. 382 probes for induced genes and 197 probes for repressed genes with statistically significant changes (p<0.01) are listed.

**Figure 9:**
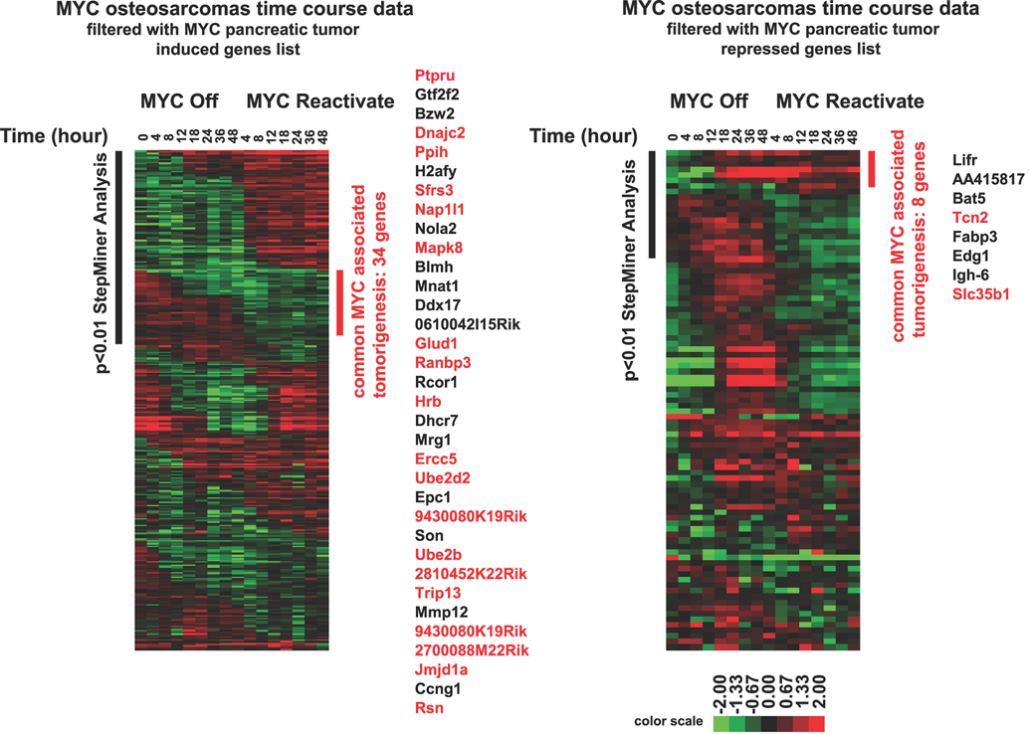
A common gene signature associated with the ability of MYC to induce tumorigenesis in murine conditional tumor models. Microarray data from the time-course experiment in MYC induced osteosarcoma was filtered with the list of tumor maintenance genes from pancreatic tumors (Figure 8). 34 genes from the induced gene list and 8 genes from the repressed gene list were identified (p<0.01) as common MYC target genes associated with MYC induced tumorigenesis in mice. Genes with E-box sequences in their promoter regions (−2000 to +2000) are labeled with red and the numbers of E-box in each gene are listed in Table S11.

We first used the StepMiner algorithm was applied to the raw data generated from these published experiments to obtain lists of genes that increase (or decrease) in expression upon tumorigenesis and decrease (or increase) in expression upon tumor regression (Figure 8 and Table S8). After StepMiner analysis, 196 and 65 unique probes were identified as induced and repressed genes respectively, which are associated with MYC-induced tumorigenesis. The osteosarcoma data set was filtered via the induced gene list or the repressed gene list generated from the pancreatic tumors. Then, we applied StepMiner analysis to identify genes that are permanently repressed or permanently induced with a p-value<0.01. By comparing microarray data from two independent MYC conditional tumor models, we found a common gene signature with 42 genes associated with MYC-induced tumorigenesis (Figure 9). Among the list of genes, there are 34 unique genes positively correlating with MYC-induced tumorigenesis and 8 unique genes negatively correlating with MYC-induced tumorigenesis in mice (Figure 9).

### Boolean Analysis of Murine MYC Signature in Human Datasets

**Figure 10:**
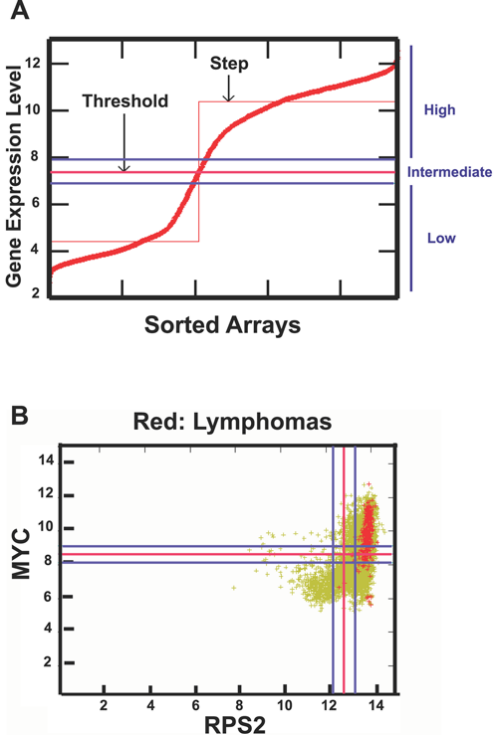
Overview of Boolean analysis of gene expression in all human microarray data with U133A format. (A) The gene expression from all the U133A arrays was first normalized and ordered from low to high. Each red mark represents one array data (total of 7,171 arrays). The StepMiner algorithm was then applied to define a threshold (the red line) and an “intermediate” region was set with a value of 0.5 around this threshold (blue lines). The expression above or below the “intermediate” region is considered as “high” or “low”, respectively. (B) A scatter plot of MYC expression (Y axis) versus RPS2 (X axis) is shown here. Each green mark represents one array data (total of 7,171 arrays). Red spots represent lymphomas (total of 273 arrays). The red lines represent the thresholds and the blue lines mark the “intermediate” region. Other examples of Boolean analysis are shown here (Figure S7).

MYC overexpression has been implicated in the pathogenesis of many types of human cancer, in particular, hematopoietic tumors [1]. To see if the gene signature we defined in murine tumor models was predictive of genes whose expression was strongly correlated with MYC between MYC and human homologs in human lymphomas, we retrieved all publicly available human microarrays (n = 7,171) in Affymatrix U133A platform. Then, we classified the expression level of each gene on each array as “low” or “high” relative to a threshold using Boolean analysis ([29] and Sahoo *et al.* RECOMB 2007 in press, see Figure 10A). We found that MYC expression is “high” in human lymphomas (204 out of 221 lymphoma cases ignoring the “intermediate” values, see Figure 10B). Figure 10B shows the gene expression scatter plot of MYC and RPS2, which are both highly expressed in lymphoma arrays (total of 273 lymphoma microarrays are highlighted with red color). We then examined to see if the expression of MYC-associated genes identified above (Figure 4 and 9) are “high” or “low” in more than 95% of the lymphoma microarrays.

**Figure 11:**
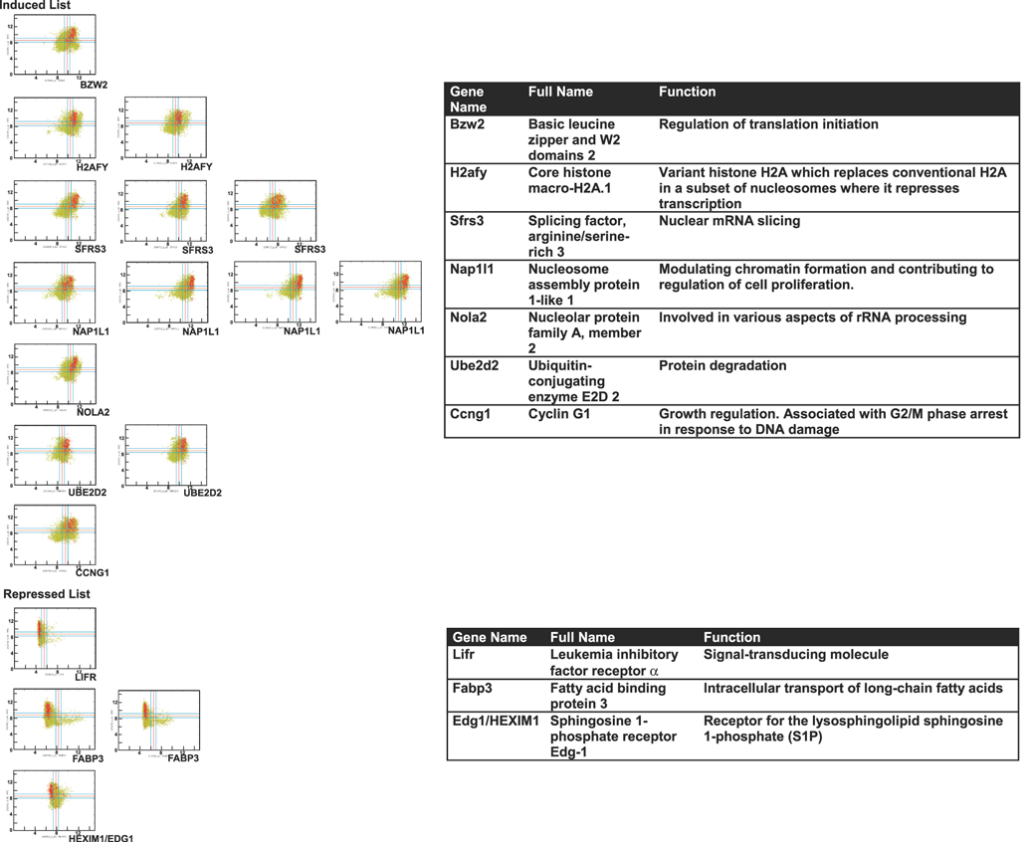
High correlation of expression between MYC and human homologs of genes associated with MYC-induced tumorigenesis in human lymphomas by Boolean analysis. Expression of human homologs of genes associated with MYC-induced tumorigenesis was examined by Boolean analysis. Scattered plots of MYC and genes from the induced list which were highly expressed in more than 95% of the lymphomas microarrays and genes from the repressed list whose expression is low in more than 95% of the lymphomas microarrays were listed here. List of genes with biological functions are listed on the right.

The Boolean analysis identified that the expression of both small and large ribosomal structural proteins is high in human lymphomas (Figures S3 and S4) as was observed in murine osteosarcomas and lymphomas (Figure 4). We further investigated if the expression of human homologs of the common gene signature from the murine microarray data is “high” or “low” in human lymphomas. 63 unique probes from the induced list (Figure 9) and 9 probes from the repressed list (Figure 9) were found in the U133A format (see Table S9). We found 14 out of 63 probes correlated with the human arrays. Genes whose expression was “high” in more than 95% of human lymphomas, whose gene names include: BZW2, H2AFY, SFRS3, NAP1L1, NOLA2, UBE2D2 and CCNG1 (p = 4.07×10^−5^, Figure 11). From the repressed list of genes 4 out of 9 probes had low expression in more than 95% of the human lymphomas, whose gene names include LIFR, FABP3 and EDG1/HEXIM1 (p = 0.03, Figure 11). We have listed al the genes identified and their associated functions (listed in the Swiss-Prot data base) (Figure 11). Many of these genes have functions that could account for MYC activity. Notably, CCNG1, LIFR and EDG1/HEXIM1 are involved in cell cycle or signaling pathways. H2AFY and NAP1l1 are involved in modulating chromatin structures. SFRS3 and NOLA2 are involved in mRNA and rRNA processing. BZW2, UBE2D2 and FABP3 are involved in metabolism such as protein or fatty acid synthesis.

Finally, we validated our results obtained by microarray analysis through quantitative real-time PCR (Figure S5). Moreover, we found that these identified genes exhibited similar patterns of changes in gene expression upon MYC inactivation in our model of MYC-induced lymphoma (Figure S6). Therefore, we have identified a subset of MYC regulated gene products that are highly correlated with the ability of MYC to maintain tumorigenesis.

## Discussion

MYC target genes have been implicated in a multitude of biological functions [16]. Many additional potential MYC targets have been identified through microarray analysis [3], [15], [21]–[26],[37]. However, it has not been easy to discern which if any of these genes are involved in the ability of MYC to initiate or maintain tumorigenesis. We have combined microarray analysis of two conditional transgenic model systems and a human comparative Boolean analysis to determine which of these identified genes most strongly correlated with MYC expression from total of 273 datasets of human lymphoma microarrays in U133A format. We also utilized ChIP to demonstrate that a large number of the genes that were permanently suppressed upon MYC inactivation exhibited changes in the ability of MYC to bind to their promoter loci. Thus, we identified a gene signature strongly correlated with the ability of MYC to maintain tumorigenesis. Our results have possible implications for why MYC induces tumorigenesis in specific cellular contexts.

To identify this gene signature, we utilized our conditional transgenic model system of MYC-induced osteosarcoma in which we have previously shown that upon MYC inactivation tumors permanently lost the ability of MYC to induce tumorigenesis [6]. Thereby, we defined an initial gene signature consisting of 2,793 unique probe sets of genes that included genes whose expression was permanently changed (Figure 3). This gene signature includes gene products that have been already implicated as MYC targets (Figure 5). Most notably, ribosomal structural proteins were strongly correlated with MYC-induced tumorigenesis in murine osteosarcomas, lymphomas (Shachaf CM et. al. submitted) and in human lymphomas. These results suggest that the ability of MYC to induce ribosomal gene products is important to its ability to initiate and maintain tumorigenesis.

Our results are consistent with a multitude of evidence suggesting that MYC can regulate ribosomal gene expression [15]. In *Drosophila*, the biological connection of MYC and ribosomal structural proteins can also be seen in the small cell-size phenotypes of both MYC mutants and ribosomal structural protein genes mutants [38]–[40]. MYC globally regulates protein synthesis through regulating expression of ribosomal RNAs, tRNAs, RNA helicases, and translation elongation factors [18],[41]. Notably, it had been shown that rate of protein synthesis was increased 3-fold in MYC-overexpressing fibroblasts compared to MYC knockout fibroblasts [42]. We confirmed that the inactivation of MYC in tumor cells resulted in a reduction of both ribosomal protein gene expression and rate of protein synthesis in murine tumor models (Figure 4). Ribosomal genes could play important function in influencing protein translation and thus in this manner influence the ability of MYC to function as an oncogene. In this regard, it is notable that a recent study in Zebra fish identified some ribosomal protein genes as tumor-suppressors [43]. Nevertheless, it is not clear how ribosomal structural protein genes function as tumor-suppressors during tumorigenesis.

Interestingly, changes in the gene expression of ribosomal structural proteins, although observed in both our model of MYC induced osteosarcoma and lymphoma, were not seen in a model of pancreatic islet cell tumors (Figure 4, 8, and [25]). Thus, it is possible that ribosomal protein genes expression play a role MYC-induced tumorigenesis only in specific types of cancer. We are reassured of the likely importance of ribosomal gene products in MYC associated tumorigenesis for we were able to confirm that MYC and ribosomal structural proteins are highly correlated in human lymphomas (Figure S4 and S5). It remains to be directly determined if these ribosomal genes are playing a role in MYC induced tumorigenesis.

Genes that we identified as most strongly correlated with MYC-induced tumorigenesis (Figure 9) in mice are involved in diverse biological processes such as transcription regulation, RNA processing, proliferation, fatty acid transport and cell signaling (Figure 11). Furthermore, some of the genes identified have been previously implicated in tumors or oncogenic signaling pathways. BLMH has been previously shown to be a MYC target [44]. UBE2d2 has been implicated as a target of the WNT signaling pathway in a microarray experiment [45]. NAP1l1 has been shown to be a tumor marker for colon cancer [46]. TRIP13 expression was highly elevated in tumor tissues [47]. Altered regulation of CCNG1 has been observed in breast cancer [48]. High expression of NOLA2 has been seen in squamous cell lung cancer [49]. Interestingly, anti-tumor effects have been observed for genes with expression reversely correlated with MYC. FABP3 has been proposed as tumor suppressor in breast cancer [50]. EDG1 has been shown to be an inhibitor for breast cancer growth [51]. Our data now suggest that BZW2, H2AFY and SFRS3, which function in translation initiation [52], chromatin structure [53], and mRNA splicing [54], respectively, may also be involved in tumorigenesis.

We were able to utilize our MYC conditional tumor models as tools to uncover genes that are strongly correlated with tumor maintenance. However, we recognize that it is very unlikely that any of the individual genes we identified are sufficient alone to explain the ability of MYC to initiate or maintain tumorigenesis. Rather it is highly likely that it is a constellation of gene expression changes that are responsible for the ability of MYC to maintain tumorigenesis.

**Figure 12:**
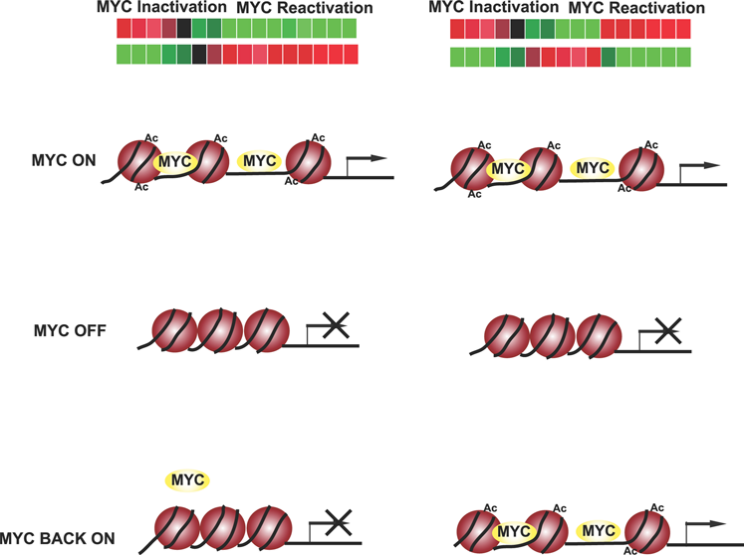
An illustration for a possible mechanism for permanent changes of gene expression upon MYC inactivation in osteosarcomas.

We can now offer a possible explanation for why the brief inactivation of MYC can result in the permanent loss of the ability of MYC to sustain tumorigenesis [6]. MYC inactivation appears to result in permanent changes in the ability of MYC to function as a transcription factor (Figure 12). Recently, we have shown that MYC inactivation induced chromatin modifications associated with cellular senescence [14]. The particular structural state of chromatin has been shown to influence the ability of MYC to bind to specific promoter loci [36]. Indeed, our results illustrate that upon MYC inactivation there were permanent changes in the ability of MYC to bind to the promoters of specific gene loci (Figure 7). It remains to be determined the mechanism of these changes in chromatin structure. One possibility is that MYC itself is contributing to changes in chromatin structure through global changes in chromatin modifications, which seems an attractive possibility based upon the work from many laboratories [14],[20],[55]. Regardless of the mechanism, our results point to the fact that the genes that MYC can regulate are different in different cellular contexts and that this appears to have a direct bearing on when MYC overexpression results in a neoplastic phenotype. We note that we could not explain all of the permanent changes in gene expression based upon differences in MYC binding to promoter loci. Thus, it is likely there are additional mechanisms by which MYC's ability to regulate gene expression has been altered.

One of the biggest challenges in understanding how MYC contributes to tumorigenesis has been to address the conundrum that MYC has both direct and indirect influence on the expression of so many different genes and these genes are involved in a multitude of biologic functions. Many of these genes may not be relevant to how MYC overexpression contributes to tumorigenesis. Here we have illustrated by using a defined transgenic mouse model that exhibits conditional tumorigenesis such that upon MYC inactivation tumor cells permanently loses a neoplastic phenotype that we can define a specific gene list that is specifically correlated with MYC's ability to maintain tumorigenesis. To perform this analysis we combined two novel methods of gene expression analysis, the StepMiner and the Boolean analysis, as a powerful strategy to perform an unbiased comparative analysis of microarray data from conditional MYC-induced tumor models and all the available published human data with Affymetrix U133A format. Our strategy may be generally useful for the identification of gene signatures associated with the ability of specific oncogenes to initiate and sustain tumorigenesis and the identification of potential new therapeutic targets for the treatment of cancer.

## Materials and Methods

### Cell Culture, Doxycycline Treatment, and Rate of Protein Synthesis

Osteosarcoma-derived cell line 1325 [6] were cultured with DMEM medium supplemented with 10% FBS, 1% Pen/Strep, L-Glutamine, and non-essential amino acids (Invitrogen). Lymphomas were cultured with RPMI medium supplemented with 10% FBS, 1% Pen/Strep, L-Glutamine and 3.96×10^−4^% of 2-mercaptoethanol (Sigma). 20ng/ml of doxycycline was added to the medium for inactivating MYC expression. Seven times, each time with 20 mls of PBS, was applied to cells to completely remove doxycycline in the medium.

For rate of protein synthesis, lymphoma-derived cell line 6780 [14] or bone tumor cell line 1325 grown in complete medium with or without doxycycline were rinsed with PBS and then replenished with DMEM (with or without doxycyline) without methionine and cysteine (Invitrogen), containing 10% dialyzed fetal calf serum (Invitrogen), 1% Pen/Strep, L-Glutamine. One hour later, cells were labeled with 30 μCi of EXPRE^35^S^35^S (PerkinElmer) per plate for 60 minutes and then washed with PBS. Cells were lysed and TCA precipitation was applied to determine the incorporation of radiolabeled amino acids. Aliquots of cell lysate were used for protein determination by DC Protein Assay (Bio-Rad). The protein synthesis rate was calculated as TCA-precipitable counts per minute divided by micrograms of protein in the same sample.

### Quantitative Real-Time PCR

cDNA were synthesized by Superscript II (invitrogen) followed by manufacture's protocol. Real-time PCR for human c-MYC (probes and primers from Applied Biosystems) and mouse GAPDH [56] were performed in ABI PRIZM analyzer. Sequences for primers for quantitative real-time are listed in Table S8.

### cDNA Labeling for Microarray Experiments

Mouse cDNA microarrays were produced at Stanford Functional Genomic Facility. cDNA labeling and hybridization were followed as previously described [57]. Briefly, mRNA from bone tumor cells were extracted by Trizol (Invitrogen) based on the protocol provided by the manufacturer. 30 μg of total RNA from bone tumor and reference RNA generated by pooling RNA from various mouse tissues were used for each microarray experiment. cDNA from bone tumor cells was labeled with Cy5-dUTP and reference cDNA was labeled with by Cy3-dUTP (Amershan) after reverse-transcription. Labeled cDNAs were concentrated by Microcon YM-30 (Millipore) before hybridizing with microarrays for 16 hours at 65°C. After hybridization, microarrays were washed and spin dry before scanned on the GenePix 40000B Array Scanner (Axon). Raw array images were analyzed using the GenePix 5 software (Axon). Microarray data was then submitted to the Stanford Microarray Database (SMD) for normalization. Data after normalization was then applied with the StepMiner algorithm to identify changes in gene expression.

### StepMiner Analysis

The StepMiner fits step functions to time-course microarray data and provides a statistical measure of the goodness of fit [29]. The steps are placed between time points at the sharpest change between low expression and high expression levels, which gives insight into the timing of the gene expression-switching event. Mathematically, steps are placed at a position that minimizes the sum of square error and an F-statistic with appropriate degrees of freedom is used to produce a p-value for the goodness of fit. The StepMiner automatically characterizes the genes in to five different groups: Up, Down, Up-Down, Down-Up and Other [29]. The genes are primarily sorted in ascending order according to the timing of their change and secondarily sorted in ascending order according to their p-values.

### Chromatin Immunoprecipitation (ChIP)

ChIP was performed based on the protocol provided in the kit with some modifications (ChIP assay kit by Upstate Biotech). Briefly, bone tumor cells were grown on the condition described above with (MYC OFF and MYC reactivated conditions) or without (MYC ON condition) doxycycline (20ng/ml). 48 hours treated with doxycycline, cells were either harvested (as MYC OFF condition) or extensively washed with PBS (see above) to remove doxycycline in the medium. 48 hours after washing, cells were harvested (as MYC reactivated).

Formaldehyde (Fisher) was added to the medium to a final concentration of 1% for cross-linking at 37°C for 10 minutes. Cross-linking was stopped by adding glycine to a final concentration of 0.125M. Cells were washed with cold PBS containing protease inhibitors (1mM PMSF, 1 μg/ml aprotinin and 1 μg/ml pepstatin A) and pelleted by centrifugation. Cell pellets were then lysed in SDS lysis buffer (1% SDS, 10mM EDTA, 50mM Tris, pH 8.1, with proteases inhibitors mentioned above). Cells were sonicated with a Branson 250 sonicator at a power setting of 3 for 3 times with 10 sec for each sonication and the cells were cooled down with ice for 1 min between each sonication. This condition of sonication yielded genomic DNA fragments with a size about 100–600 base pairs. Samples were then immunoprecipitated with c-MYC antibody (2 μg of N262 from Santa Cruz Biotech) followed the protocol provided by the kit (Upstate Biotech). DNA samples from the ChIP experiments were applied for quantification by Real-time PCR (ABI PRISM 7900 HT) with SYBR green. Promoter sequences (−2000 to +2000 relative to the transcription start sites) of murine MYC targets were retrieved from UCSC genome browser and primers flanking the E-box were designed by Primer3 (http://frodo.wi.mit.edu/) (Table S10).

### Boolean Analysis of Human Microarray Data

Data from 7,171 publicly available raw Affymetrix U133A human microarrays were collected from the Gene Expression Omnibus (GEO) [58] and normalized together using the RMA algorithm [59],[60]. Thresholds were assigned for each probe set by first sorting the expression values for that probe set on all arrays in ascending order, and then fitting a step function to the data using the StepMiner. This approach places the threshold cutoff at the largest jump from low values to high values. In the case where the gene expression levels are evenly distributed from low to high, the threshold cutoff tends to be near the mean expression level. If the assigned cutoff for a gene is t, expression levels above *t* + 0.5 are classified as “high,” expression levels below *t*−0.5 are classified as “low,” and values between *t* −0.5 and *t*+0.5 are classified as “intermediate” (Sahoo *et al.* RECOMB 2007 in press). Two hundred and seventy three different human Lymphoma microarray experiments were identified using a simple string search “Lymphoma” in the GEO description of the experiment. Genes that are “high” or “low” in more than 95% of the Lymphoma experiments were automatically discovered. Human homologs of genes which were associated with MYC-induced tumorigenesis in mice were selected for this manuscript.

## Supporting Information

Figure S1 Western blot for MYC protein expression upon MYC inactivation and reactivation. Western blots for anti-MYC and α-tubulin antibodies [14] upon MYC inactivation and reactivation are shown here.
(1.21 MB EPS)

Figure S2 Induced and repressed patterns of gene expression upon MYC inactivation in osteosarcomas identified by the StepMiner algorithm. MYC OFF arrays (MYC inactivation for 0, 4, 8, 12, 18, 24, 36, 48 hours) were selected for the StepMiner analysis to retrieve significant changes as the “induced” and “repressed” patterns. Statistically significant changes (p<0.01) after StepMiner analysis are shown here. Genes previously shown to be associated with senescence are highlighted.
(1.75 MB EPS)

Figure S3 High expression of both MYC and small ribosomal structural proteins in human lymphomas by Boolean analysis. MYC expression (Y axis) and small ribosomal structural protein expression (X axis) were plotted for all the published human microarrays in U133A format (green marks). Red lines represent the cutoff thresholds and blue lines mark the “intermediate” regions. Green marks represent all human arrays and red marks represent arrays from lymphoma tissues.
(29.83 MB EPS)

Figure S4 High expression of both MYC and large ribosomal structural proteins in human lymphomas by Boolean analysis. MYC expression (Y axis) and large ribosomal structural protein expression (X axis) were plotted for all the published human microarrays in U133A format (green marks). Red lines represent the cutoff thresholds and blue lines mark the “intermediate” regions. Green marks represent all human arrays and red marks represent arrays from lymphoma tissues.
(35.18 MB EPS)

Figure S5 Validation of mRNA expression of gene signature in MYC-induced osteosarcomas by quantitative real-time PCR. Quantitative real-time PCR with primers specific for genes associated with MYC induced tumorigenesis were applied to cDNA samples upon MYC inactivation for 0, 8, 24, 48 and MYC reactivation for 8, 24 and 48 hours in MYC induced osteosarcomas to validate the mRNA expression. mRNA expression was normalized by r-tTA expression. Relative expression levels were shown here (MYC inactivation for 0 hour was reset to 1).
(3.99 MB EPS)

Figure S6 Validation of mRNA expression of gene signature in MYC-induced lymphomas by quantitative real-time PCR. Quantitative real-time PCR with primers specific for genes associated with MYC induced tumorigenesis were applied to cDNA samples upon MYC inactivation for 0, 6, 12, 24, 48 hours in MYC-induced lymphomas. mRNA expression was normalized by r-tTA expression. Relative expression levels were shown here (MYC inactivation for 0 hour was reset to 1).
(3.08 MB EPS)

Figure S7 Examples of Boolean analysis for expression of BUB1B vs. CCNB2 and XIST vs. RPS4Y1 in the U133A format. Both BUB1B and CCNB2 were found to be expressed in a cell cycle dependent manner [61],[62]. Boolean analysis for all the arrays in the U133 A format indicates a strong correlation of expression between the two genes (left panel). XIST is expressed from the inactivated×chromosomes [63],[64]. RPS4Y1 is a transcript from the Y chromosomes [65]. Boolean analysis for all the arrays in the U133 A format indicates the exclusive expression pattern between the two genes (right panel).
(1.86 MB EPS)

Table S1 List of LUIDs, gene names and Genbank accession numbers of induced and repressed genes upon MYC inactivation.
(0.24 MB XLS)

Table S2 List of LUIDs, gene names and Genbank accession numbers of genes permanently repressed (PR), permanently induced (PI), reversibly repressed (RR), and reversibly induced (RI) upon MYC inactivation.
(0.37 MB XLS)

Table S3 GO term analysis of genes permanently induced or repressed upon MYC inactivation in osteosarcoma. Permanently induced (PI) genes (expression went up and stayed up) and permanently repressed (PR) genes upon MYC inactivation was analyzed by GO Term to identify possible representative biological processes in each time points along the time-course for MYC inactivation. Statistically significant (p<0.01) biological processes in each step are listed here (step 0: between MYC OFF 0 and 4 hours, step 1: between MYC OFF 4 and 8 hours, step 2: between MYC OFF 8 and 12 hours, step 3: between MYC OFF 12 and 18 hours, step 4: between MYC OFF 18 and 24 hours, step 5: between MYC OFF 24 and 36 hours, step 6: between MYC OFF 36 and 48 hours).
(0.04 MB DOC)

Table S4 Gene Ontology (GO) term analysis for genes with PI patterns upon MYC inactivation. Permanently induced (PI) genes (expression went up and stayed up) upon MYC inactivation was analyzed by GO Term to identify possible representative biological processes in each time points along the time-course for MYC inactivation. Statistically significant (p<0.01) biological processes in each step (step 0: between MYC OFF 0 and 4 hours, step 1: between MYC OFF 4 and 8 hours, step 2: between MYC OFF 8 and 12 hours, step 3: between MYC OFF 12 and 18 hours, step 4: between MYC OFF 18 and 24 hours, step 5: between MYC OFF 24 and 36 hours, step 6: between MYC OFF 36 and 48 hours).
(0.06 MB XLS)

Table S5 Gene Ontology (GO) term analysis for genes with PR patterns upon MYC inactivation. Permanently repressed genes (expression went down and stayed down) upon MYC inactivation was analyzed by GO Term to identify possible representative biological processes in each time points along the time-course for MYC inactivation. Statistically significant (p<0.01) biological processes in each step (step 0: between MYC OFF 0 and 4 hours, step 1: between MYC OFF 4 and 8 hours, step 2: between MYC OFF 8 and 12 hours, step 3: between MYC OFF 12 and 18 hours, step 4: between MYC OFF 18 and 24 hours, step 5: between MYC OFF 24 and 36 hours, step 6: between MYC OFF 36 and 48 hours).
(0.08 MB XLS)

Table S6 List of gene names with the PR and PI patterns for three previously published reports regarding direct MYC targets [33]–[35] and the RR pattern for one previously published reports regarding direct MYC targets [35]. Murine homologs of human direct MYC targets [33]–[35] were retrieved and used as a list to filter the bone tumor microarray data. Genes with the PR, PI and RR patterns which are statistically significant after the StepMiner analysis are shown here.
(0.09 MB XLS)

Table S7 Raw data of ChIP experiments. ChIP for MYC binding to E-box regions were examined for ribosomal structural proteins (figure 4) and genes with the PR pattern and RR pattern expression whose homologs shown to be direct MYC targets before (figure 6 and [35]). Real-time PCR was applied to measure MYC binding to the E-box regions in the promoter of these MYC targets upon MYC ON, MYC inactivated for 48 hours, and MYC reactivated for 48 hours. Percentage of DNA brought down by ChIP was calculated as shown before [19].
(0.21 MB XLS)

Table S8 List of gene names which were induced (repressed) in tumorigenesis (MYC activation) and repressed (induced) in tumor regression (MYC inactivation) in MYC-induced murine pancreatic tumors. The StepMiner algorithm was applied to the raw microarray data generated from the pancreatic tumors upon MYC activation and MYC inactivation [25]. Induced and repressed gene lists with p<0.01 after the StepMiner analysis are shown here.
(0.04 MB XLS)

Table S9 List of Affymetrix probe IDs of human homologs of genes associated with MYC-induced tumorigenesis in mice.
(0.02 MB XLS)

Table S10 List of primer sequences and promoter sequences for quantitative real-time PCR and ChIP.
(0.65 MB XLS)

Table S11 List of numbers of E-box sequences in promoter regions (−2000 to +2000) of common murine MYC target genes. Promoter sequences were retrieved for common murine MYC target genes shown in figure 9 and the numbers of E-box sequences in these regions are shown here.
(0.02 MB XLS)
