# Correction: Combined Analysis of Murine and Human Microarrays and ChIP Analysis Reveals Genes Associated with the Ability of MYC To Maintain Tumorigenesis

**DOI:** 10.1371/annotation/a0e06cef-a7e4-4ec9-9f35-9df5e50bf7a2

**Published:** 2013-10-15

**Authors:** Chi-Hwa Wu, Debashis Sahoo, Constadina Arvanitis, Nicole Bradon, David L. Dill, Dean W. Felsher

The first two authors, Chi-Hwa Wu and Dabashis Sahoo, should be noted as contributing equally to this work.

