# Supplementary material for: Combined Analysis of Murine and Human Microarrays and ChIP Analysis Reveals Genes Associated with the Ability of MYC To Maintain Tumorigenesis
Source: PLoS Genet. 2008 Jun 6;4(6):e1000090. doi: 10.1371/journal.pgen.1000090 (PMC2390767; doi:10.1371/journal.pgen.1000090)
Supplement: Table S3 — GO term analysis of genes permanently induced or repressed upon MYC inactivation in osteosarcoma. Permanently induced (PI) genes (expression went up and stayed up) and permanently repressed (PR) genes upon MYC inactivation was analyzed by GO Term to identify possible representative biological processes in each time points along the time-course for MYC inactivation. Statistically significant (p<0.01) biological processes in each step are listed here (step 0: between MYC OFF 0 and 4 hours, step 1: between MYC OFF 4 and 8 hours, step 2: between MYC OFF 8 and 12 hours, step 3: between MYC OFF 12 and 18 hours, step 4: between MYC OFF 18 and 24 hours, step 5: between MYC OFF 24 and 36 hours, step 6: between MYC OFF 36 and 48 hours). (0.04 MB DOC) [file pgen.1000090.s010.doc]

Table S3

| **Permanently Induced (PI)** | | **Permanently Repressed (PR)** | |
| --- | --- | --- | --- |
| Step 0 |  | Step 0 |  |
| Step 1 |  | Step 1 |  |
| Step 2 |  | Step 2 | mitochondrion ribonucleoprotein complex |
| Step 3 | extracellular matrix | Step 3 |  |
| Step 4 |  | Step 4 | platelet-derived growth factor receptor signaling pathway |
| Step 5 |  | Step 5 | mitochondrion  RNA binding |
| Step 6 |  | Step 6 |  |
| Step 7 |  | Step 7 | proteasome core complex  proteasomecomplex  mitochondrion  ribonucleoprotein complex |
| Step 8 | regulation of transcription, DNA-dependent  protein binding  nucleus  metal ion binding | Step 8 | ribosome  structural constituent of ribosome  NADH dehydrogenase (ubiquinone) activity  mitochondrionmitochondrial inner membrane ribonucleoprotein complex |
| Step 9 |  | Step 9 |  |
| Step 10 |  | Step 10 |  |
| Step 11 |  | Step 11 |  |
| Step 12 |  | Step 12 |  |
| Step 13 |  | Step 13 |  |
| Step 14 |  | Step 14 |  |
